# Numerical and Experimental Studies of Mechanical Performance and Structural Enhancement of Industrial Building SSMRs

**DOI:** 10.3390/ma15093163

**Published:** 2022-04-27

**Authors:** Koochul Ji, Hyok Chu Choi, Kyungrok Kwon, Jung Sik Kong

**Affiliations:** 1 Global Loss Control Center, Samsung Fire & Marine Insurance Co., Ltd., Seoul 04523, Korea; koochul.ji@samsung.com (K.J.); hyok714@korea.ac.kr (H.C.C.); 2 Department of Civil, Environmental and Architectural Engineering, Korea University, Seoul 02841, Korea; kkr929@korea.ac.kr

**Keywords:** standing seam metal roof, roof structure enhancement, industrial building roof, lab-scale experiment, failure wind speed

## Abstract

In response to the increasing demands of high-technology industrial buildings, renovated standing seam metal roofs (SSMRs) are widely used in the construction of such buildings due to their superior performance regarding heat insulation and waterproofing. However, studies to identify realistic mechanical performance and structural defects in newly applied SSMRs are still limited due to their recent development. In our previous full-scale experiment, the ultimate failure of the roof under wind pressure corresponded to mid-clip failure rather than end clip failure and seam separation; therefore, in this study, the lab-scale experimental programs mainly focused on the mid-clip and the metal roof sheet. Here, the plastic saddle type of the SSMR was chosen as the lab-scale experiment specimen under various loading speeds and angled plastic saddle conditions. The JC material properties were calibrated against experimental results and simulated to predict the dynamic failure response of SSMRs. An additional experimental study was conducted to identify the effect of strengthening SSMRs with wind clips, which showed that 20.77% of the peak load was enhanced after reinforcing the SSMR with wind clips. On the basis of this result, the failure wind speed was computed according to ASCE 7–10 standards with the assumption of a wind clip installed on the corner and edge of the roof panel, indicating that the failure wind speed increased with the wind clip by about 6 to 7 m/s. The current research results suggest a methodology for enhancing the structural performance of renovated industrial building SSMRs.

## 1. Introduction

Metal roof panels are widely used as the roofing systems of industrial buildings, as they have the advantages of a shorter construction period, lower cost, and a lower weight that does not require large supporting structures. However, a typical damage pattern in which roof panels are torn off due to wind uplift loading from strong wind is common. Recently, there has been growing concern that tropical cyclones accompanied by extremely strong winds and heavy rain cause complex and chain-like occurrences of damage to industrial buildings, leading to business interruptions due to damage to critical and essential facilities or stored products [1]. To alleviate the damage induced by strong winds and heavy rain, the standing seam metal roof (SSMR) design was recently developed as a roof panel type reflecting the requirements of high-tech industrial buildings that require waterproofing and insulation performance. However, the studies on SSMRs are insufficient in terms of proven structural performance, installation, and management standards. As a high-technology industrial building requires a multifunctional roof system, manufacturers have been paying attention to developing additional structural components for heat insulation and waterproofing regardless of the vulnerability to strong winds [2]. Therefore, the potential risk of major damage from strong winds is growing. Previous studies on metal roof systems are still limited and mostly focus on the existing through-fastened type (TFMR) or the earlier single roofing sheet model of SSMRs for industrial buildings [3,4,5,6]. In addition, researchers have focused on the influence of wind loads on the vulnerability of a roof system but have paid less attention to the performance level and failure modes of roof systems, which are critical for engineering and risk management [7,8,9,10].

**Figure 1:**
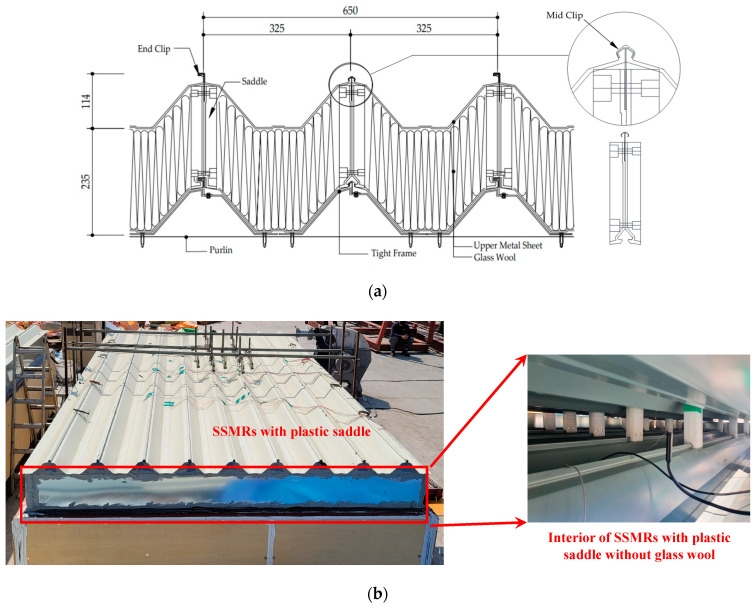
Tested SSMRs with a plastic saddle: (**a**) schematic design (unit: mm), (**b**) exterior–interior view.

In order to establish an engineering standard applicable to actual practice, it is necessary to identify the failure mode of SSMRs through an experimental method that is close to reality in order to find the component that dominates the failure and to derive a structural performance standard. In particular, the clips, which are the main components of an SSMR, have rate-dependent material properties, and differences in the structural performance may occur due to the differences in the installation state according to the type of saddle. For instance, the effect of the dynamic loading of construction materials has been studied by many researchers [11,12,13,14]. Many previous studies confirmed that the stiffness, yield, and tensile strength tend to increase when the loading rate is high in tensile tests of steel materials [15,16,17]. For instance, Meyer and Abdel-Malek conducted a comparative study on the effect of the loading rate and found that the behavior of the tensile test was affected more by the loading rate than the compression [18]. In order to accurately predict the behavior of materials in computational analysis, it is necessary to precisely determine and use the properties of its materials [19,20]. Therefore, many studies have been conducted to determine the strain-rate-related parameters of steel materials for several material behavior models that consider the strain rate behavior of construction materials. Jang and Yoo proposed a method for determining the component model coefficients of materials by linking experiments and analyses such as tensile tests for various velocities and analyses of tensile tests for metal materials [21]. Damatty and Rahma developed a finite element model by applying the spring constant estimated by a component experiment program, including the clip and seam components of SSMR. However, this model has the limitation that it cannot be applied to renovated SSMRs including a saddle and cannot consider the dynamic response of an SSMR [22]. Therefore, detailed experimental studies for the components of SSMRs and numerical models that can reflect the realistic dynamic behavior of SSMRs are needed to determine their mechanical performance. Previously, we conducted a field-scale experimental test in accordance with ASTM E1592 regulations to understand the structural performance and dominant failure mode of renovated SSMRs as shown in Figure 1 [23]. The experimental results showed that seam separation induced by mid-clip rupture was the predominant mode of failure [2]. Therefore, it was verified that the behavior of the mid-clip plays a significant role in the overall performance of SSMR components under wind uplift pressure. Furthermore, the importance of such studies for sustainability and strengthening infrastructure has been widely expressed because of an increase in the number of deteriorated infrastructures [24,25,26]. However, endeavors to strengthen renovated SSMRs are still limited due to their recent development, and the effect and performance of reinforcing the material known as the wind clip have not been proven yet, even though it has been widely installed in various high-technology industrial buildings. To assess the mechanical performance of mechanically strengthened SSMRs by wind clips, wind clips that were widely employed in the established SSMR by manufacturers of SSMR and wind clips were chosen to obtain the mechanical response with and without the wind clips.

The objective of this research was to establish structural performance standards that are applicable to engineering practice through lab-scale tests on components corresponding to the main failure mode observed in full-scale tests and to develop an analysis model that can predict the dynamic response under various loading conditions and structural defects. In this study, lab-scale tests under various loading speeds and geometric profiles to identify the sustainability of their structures were conducted to assess the performance of mid-clips and the components of SSMRs that are dominant in failure processes. Furthermore, finite-element simulations were conducted to account for the mechanical response of SSMRs on the basis of the Johnson–Cook (JC) strength model, which were widely employed for metallic materials [19,20]. The JC strength parameters of the mid-clip were calibrated with measured experimental results. This model was successfully implemented to predict the failure response of the mid-clip under various loading conditions. Based on the lab-scale tests with SSMRs strengthened by wind clips, the overall experimental and analytical results of renovated SSMRs were addressed to recognize the failure wind speed after reinforcing the corners and edges of the roof system.

## 2. Experimental Program

### 2.1. Materials

According to a previous study [2], the major failures of field-scale SSMRs were mainly observed at the mid-clips rather than end clips and seam separations. In this study, lab-scale SSMRs with a plastic saddle and mid-clips were prepared for the experimental program. Details on the material characteristics and the configuration of the roof panel system’s components are listed in Table 1. To quantify the performance of the mid-clips, each type of roof panel system was fabricated as a metal roofing sheet, mid-clip, saddle, and tight frame. Metal roofing sheets were cut with dimensions of 325 × 500 mm with 0.7 mm thickness to reproduce the uplift load and boundary conditions (distances between saddles) conducted on field-scale SSMR tests.

### 2.2. Experimental Set-Up

**Figure 2:**
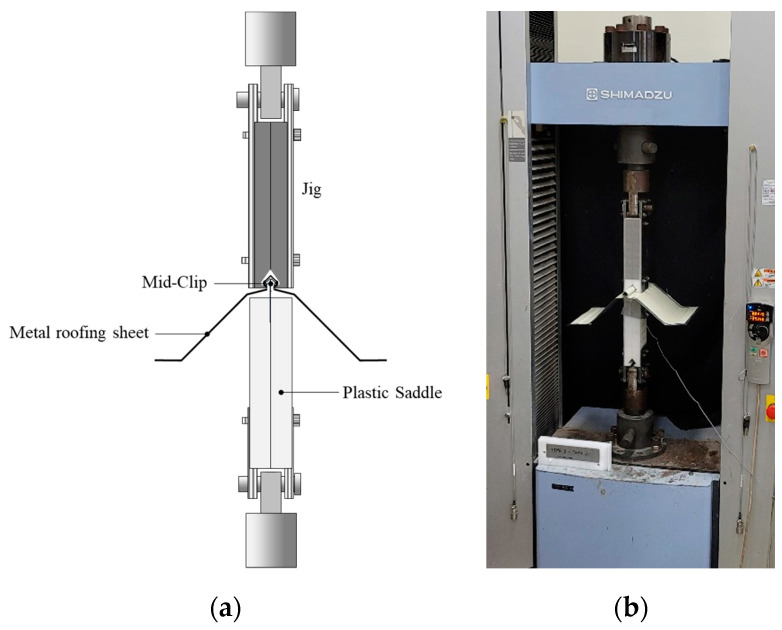
(**a**) Schematic design; (**b**) experimental set-up of the SSMR lab-scale test.

The pull-out experimental test set-ups for the lab-scale SSMRs were prepared to identify the mechanical response of mid-clips under fully fabricated conditions by considering the effect of loading velocity and saddle incline. The experimental set-up of the lab-scale test was carried out at the KICT (Korean Institute of Civil Engineering and Building Technology) in South Korea. A Universal Testing Machine (UTM) was used to perform the pull-out test with a custom designed jig and fixture to maintain the experimental boundary conditions of the metal roofing sheet and tight frame, as shown in Figure 2. Three rounds of the lab-scale experiment were conducted under various loading velocities, plastic saddle angles, and clamps to reinforce the SSMRs. The UTM had a 300 kN loading capacity with a permissible speed range of 0.005~500 mm/min. A number of constant speeds were applied to obtain the dynamic mechanical response of the mid-clip. To monitor the response of the tested metal roofing sheet and mid-clip, the load and displacement data were measured using a universal testing machine.

### 2.3. Results of the SSMR Lab-Scale Test

**Figure 3:**
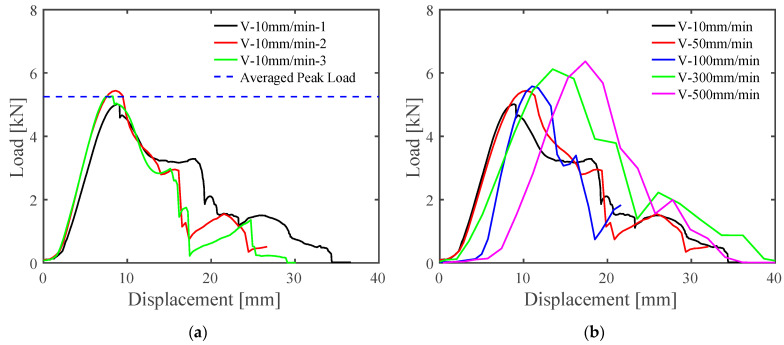
Experimental results for load vs. vertical displacement behavior (**a**) at 10 mm/min speed and (**b**) at various speed loads from 10 mm/min to 500 mm/min.

The laboratory SSMRs tests were performed at various speeds that ranged across 10 mm/min, 50 mm/min, 100 mm/min, 200 mm/min, 300 mm/min, and 500 mm/min. The applied speed and vertical displacement were obtained simultaneously at the same sampling frequency during the pull-out tests. The failure modes of the laboratory SSMR tests were observed close to the mid-clip instead of at the plastic saddle and metal roofing sheet. The results of the SSMR lab-scale tests at 10 mm/min velocity are shown in Figure 3a. The results depict the elastic deformation until reaching peak load and softening behaviors. The fluctuation of the softening regime is observed due to the rupturing of each wingtip at the mid-clip. The averaged peak load of the SSMR lab tests at 10 mm/min was 5.251 kN. Further laboratory tests were conducted at various applied velocities, as illustrated in Figure 3b. The peak load for the SSMR lab-scale tests changed with the applied velocity. The peak load and displacement at peak load changed with the applied speed. This indicated the rate dependency of the mid-clip on the SSMR specimens.

**Figure 4:**
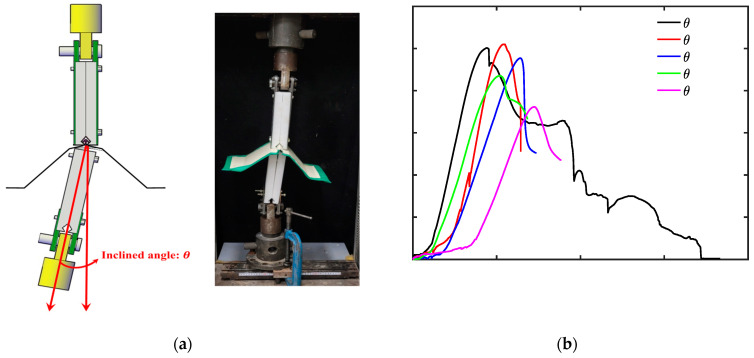
Experimental set-up for the inclined plastic saddle tests. (**a**) Schematic design, (**b**) load vs. displacement curves at various saddle angles.

Due to increases in insulation and waterproofing performance in industrial buildings, plastic saddles with increased heights were widely adopted in order to insert insulating materials between the metal roofing sheets in the newly adopted SSMRs. Furthermore, on the basis of previous field-scale experimental tests, it was observed that numerous plastic saddles were inclined during the assembly of the plastic saddle and metal roofing sheets in an SSMR system. To understand the relationship between mechanical performance of the lab-scale SSMR and the inclination of the plastic saddle and mid-clip, additional tests were carried out under changes in the plane rotation of the plastic saddle and mid-clip, as illustrated in Figure 4a with an applied velocity of 10 mm/min for all tests. The results of the peak load vs. plastic saddle angle are shown in Figure 4b. The results of the inclined plastic saddle SSMR experiment showed a decrease in the peak load with an increase in the incline angle from 0° to 1.72°, 2.58°, 3.44°, and 4.30°. It was recognized that the peak load decreased monotonically with an increase in the incline angle from 0° to 1.72°, 2.58°, and 3.44°. When the incline angle reached 4.30°, a drastic decrease in peak load was observed compared with the tests without an incline, which implies that the inclination of the plastic saddle has a crucial influence on the overall structural instability of SSMRs. The failure load and the corresponding applied speeds and saddle angles are summarized in Table 2. The overall mechanical performance of the lab-scale SSMR tests are presented as the average failure load. The results showed the clear relationship of the loading speed and saddle angle with SSMR performance.

## 3. Finite Element Analyses

### 3.1. Simulation Details

**Figure 5:**
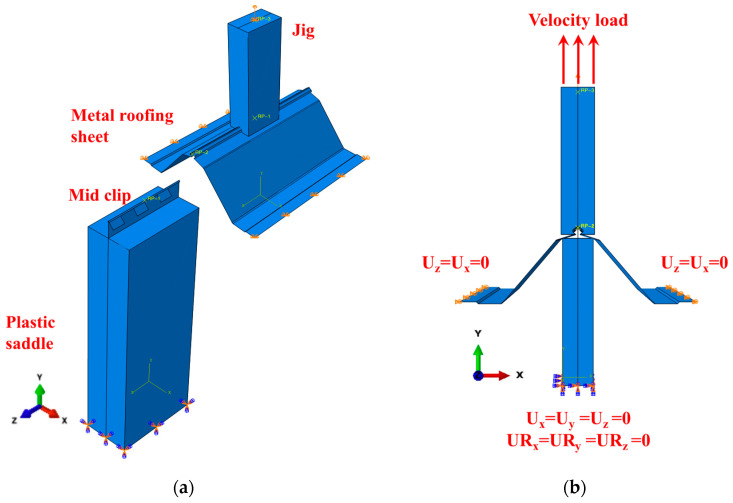
Finite element model of (**a**) the SSMR components and (**b**) the boundary conditions.

The commercial software Abaqus was used to simulate mid-clip rupture subjected to wind uplift load. The numerical simulations incorporated the mid-clip, plastic saddle, and metal roofing sheet, which had the same dimensions as the experimental specimens. The lab-scale experimental tests were simulated using a Johnson–Cook (JC) phenomenological strength model with material failure behavior to predict the strength and failure of metals. The elastic modulus and Poisson’s ratio of steel materials were determined as 200 GPa and 0.28 for all the following simulations. The Johnson–Cook model has been widely used in previous studies to predict the flow stress behavior of steels [19,27,28,29,30]. The stress and strain relationships of the JC model can be described as follows:

$$\sigma =\left(A+B\varepsilon_{pl}{}^{n}\right)\left(1+Cln\dot{\varepsilon_{*}}\right)$$

where σ and $\varepsilon_{pl}$ are the equivalent stress and the equivalent plastic strain, respectively. *A*, *B*, *C*, and *n* are the yield stress constant, the strain hardening constant, the strengthening coefficient of strain rate, and the strain hardening coefficient, respectively. The JC model equation used in this study characterized the strain hardening and the strain rate strengthening. In the model, $\dot{\varepsilon_{*}}$ can be represented as:

$$\dot{\varepsilon_{*}}=\{\begin{array}{cc} 1 & for\;\;\;\;\;\;\;\dot{\varepsilon_{pl}}<\dot{\varepsilon_{ref}}\;\\ \dot{\varepsilon_{pl}}/\dot{\varepsilon_{ref}} & for\;\;\;\;\;\;\;\dot{\varepsilon_{pl}}\;\geq \;\dot{\varepsilon_{ref}}\; \end{array}$$

where $\dot{\varepsilon_{*}}$ and $\dot{\varepsilon_{ref}}$ are the nondimensional strain rate constant and the reference strain rate. On the basis of previous studies of JC parameters, FE simulations of lab-scale SSMR tests were examined to optimize and validate the results against the experimental results. The model parameters of the JC model which were calibrated against the load and displacement response of the experimental test results are listed in Table 3. Material constants of *A*, *B*, $\dot{\varepsilon_{ref}}$, and *n* of 75 MPa, 210 MPa, 0.05651 s^−1^, and 0.5176, respectively, were obtained from previous studies on medium carbon steel for the JC model [29,30,31,32]. The strain rate strengthening coefficient, *C*, was estimated on the basis of the numerical calibration against the SSMR experimental results with respect to the peak load response. To simulate the experimental specimens and boundary conditions of SSMRs in finite element models, the plastic saddle, metal roofing sheet, and jig were prepared with the same geometry and 3D volume elements. A four-node shell element with six degrees of freedom at each node was used to model the mid-clip. The interface between the mid-clip and the metal roofing sheet was modeled with a hard contact (non-penetrability condition), with a friction coefficient of 0.2 on the assumption of interface friction between coated steels [33]. For the case of mesh sensitivity on FE analysis, lab-scale FE simulations were mainly conducted with a 4 mm mesh size by considering the computational cost on the basis of mesh sensitivity tests. The vertical velocity load was applied on the top of the jig at various loading speeds. To avoid the rotation of the metal roofing sheet during the numerical simulations, the displacement of the end edge of the metal roofing sheet was constrained in terms of the *x*- and *z*-axis, as shown in Figure 5. The displacement and rotation of the bottom plastic saddle was fixed on the *x*-, *y*-, and *z*-axes as the simulation’s boundary conditions.

### 3.2. Results of the Lab-Scale Test

**Figure 6:**
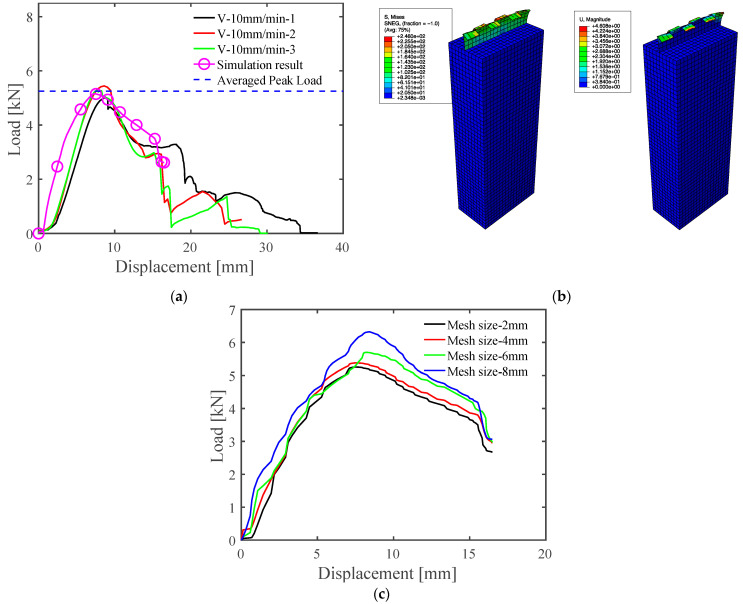
Results of the lab-scale SSMR tests: (**a**) displacement vs. load of the lab-scale test and FE result; (**b**) numerically predicted von Mises stress and displacement; (**c**) numerically predicted displacement vs. load of the FE results with different mesh sizes.

To verify the proposed JC model of the lab-scale SSMR, the numerical predictions were compared with the experimental results for various loading speeds and saddle angles. First, the experimental results obtained from the quasi-static SSMR results at the 10 mm/min loading speed were used for validation of the JC model against the load–displacement curves and failure modes predicted by the finite element analysis. A comparison of three experimental load–displacement curves with those predicted by the JC model for which the model parameters were optimized from the load–displacement results for inverse calibration is depicted in Figure 6a. The FE load–displacement curve clearly shows a correlation with the experimental results, as their maximum peak load deviation was between 1% and 6.3%. Figure 6b presents the distribution of von Mises stress and displacement for the plastic saddle and mid-clip at a 10 mm/min loading speed. The further numerical analysis was also analyzed in terms of the sensitivity of the model of SSMR to different mesh sizes (2, 4, 6, 8 mm). The predicted similar load–displacement curves are shown in Figure 6c.

**Figure 7:**
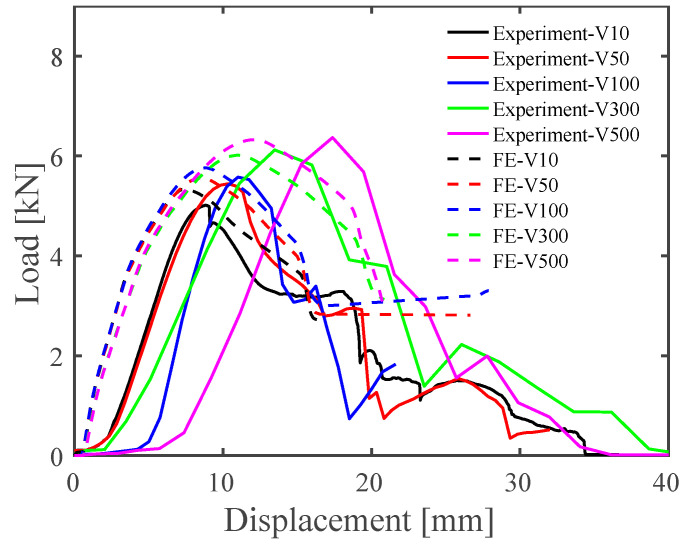
Comparison between the FE results and experimental results regarding load vs. displacement at various speeds.

Additionally, the effect of loading velocity on the load–displacement curves is shown in Figure 7, compared between the experimental results and FE results. As the loading speed increased, the peak loads of the FE simulations gradually increased from 10 mm/min to 500 mm/min. Drastic increases in the peak load and strain hardening were predicted at applied loading speeds surpassing 100 mm/min in both the experimental and the numerical studies. The overall load–displacement curves under different loading speeds derived from the JC model simulations illustrated good agreement with the experimental results. On the other hand, a discrepancy in the displacement at the peak load between the experiment and the simulation was seen over loading speeds of 100 mm/min. The maximum loads predicted by the FE simulations at 10 mm/min, 50 mm/min, 100 mm/min, 300 mm/min, and 500 mm/min were 5.332 kN, 5.559 kN, 5.761 kN, 6.280 kN, and 6.325 kN, respectively.

## 4. Discussion

**Figure 8:**
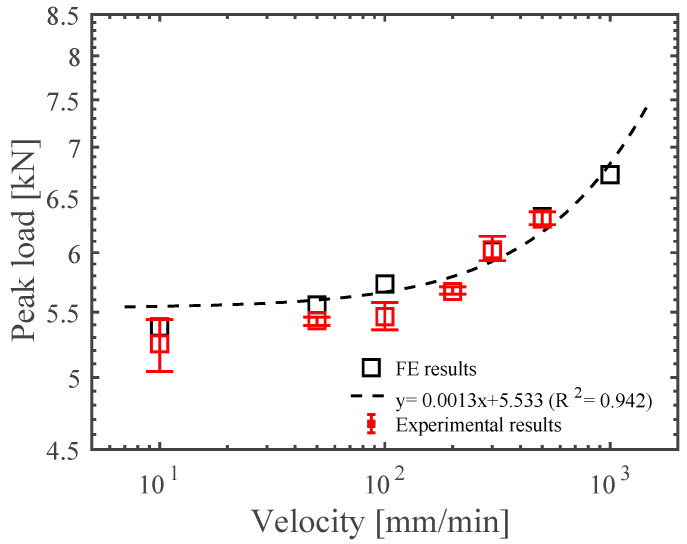
Comparison of the FE results, fitting equation, and experimental results regarding peak load vs. velocity.

Many studies have focused on vulnerability analyses of steel roofing structures on the basis of experimental and analytical studies. In this study, the mechanical performance of newly developed industrial building SSMRs was investigated under different conditions of loading velocity and saddle angle, which can induce the mechanical response of SSMRs. Furthermore, the JC material parameters of the SSMR’s mid-clip were calibrated against the experimental results. Based on these calibrated JC parameters, various FE simulations were conducted at loading velocities of 10 mm/min to 1000 mm/min. By utilizing the FE simulation, we obtained the fitted equation from the data of the failure peak load under various loading speeds, as shown in Figure 8. The fitting equation and the correlation factor R^2^ were predicted as y = 0.0013x + 5.533 and 0.942, as shown in Figure 8, which allowed us to predict the peak load response at loading velocities over 1000 mm/min. The experimental results of peak load showed good agreement between the FE results of peak load and the fitting equation. 

**Figure 9:**
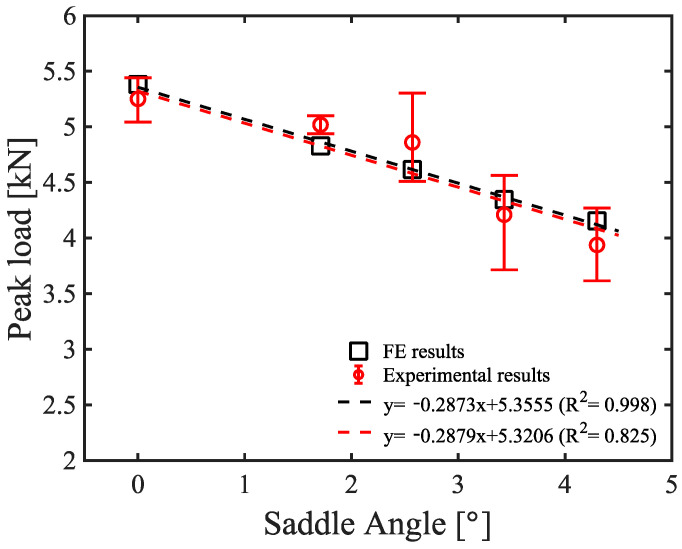
Comparison of the FE results, fitting equation, and experiment results regarding peak load vs. saddle angle.

In addition to the study on the mechanical dynamic response of SSMRs under various velocity loading conditions, an SSMR with an inclined plastic saddle and mid-clip was simulated in the finite element model as a JC material model. An experimental program, described in Figure 4 in the previous section, was duplicated by the proposed FE model for further comparison and validation. The relationship between the saddle angle and the peak load at 10 mm/min loading speed is shown in Figure 9 for both the experimental and the finite element results. The experimental results of peak load showed a gradual decrease with an increase in the saddle angle due to the stress concentrations induced by the wing of the mid-clip. Although the peak loads of the experimental tests were measured as having large variation as the saddle angle increased over 2 degrees, the experimental results of mean peak load were in good agreement with the FE results.

**Figure 10:**
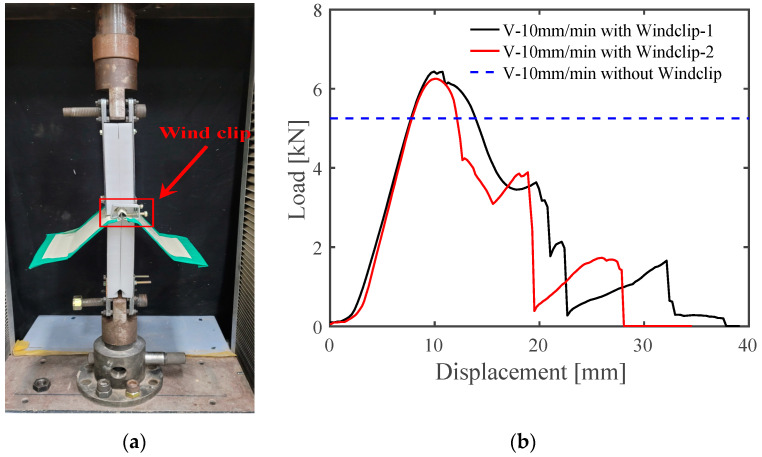
Experimental set-up for the strengthened lab-scale tests. (**a**) Schematic design; (**b**) load vs. displacement curves with wind clips.

In addition to experimental and numerical studies of the factors influencing the mechanical performance of SSMRs, the enhancement of the structural performance of SSMRs was investigated by using wind clips on the roofing sheet. The load and vertical displacement were measured in the same manner as in the experiments without a strengthened SSMR. The test set-up is shown in Figure 10a. The wind clip was installed at the location of the mid-clip to restrain the lateral displacement of the roofing sheet. Figure 10b illustrates the load vs. vertical displacement curves of the SSMRs with and without wind clip reinforcement. According to the load vs. displacement curves, the failure loads of the SSMR reinforced by wind clips were 6.433 kN and 6.250 kN. The average peak load value of the nonreinforced SSMR was 5.251 kN, shown as the blue horizontal dashed line in Figure 10b. The averaged failure load in the experiments with wind clips was 6.342 kN, which is an increase of about 20.77% compared with the average failure load of nonreinforced SSMRs, as shown in Table 4.

**Figure 11:**
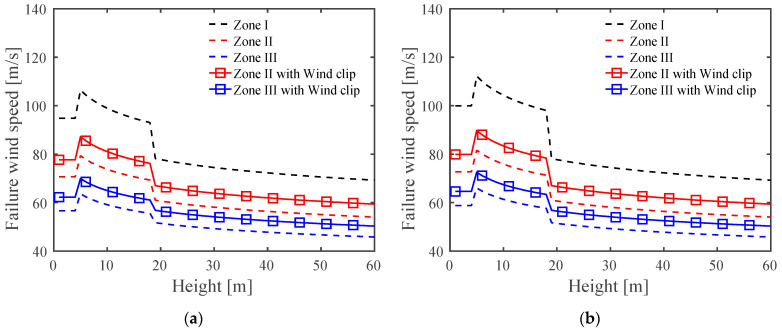
Failure wind speed vs. SSMR height increase of three roof zones with and without wind clip enhancement. (**a**) Roof angles less than 7°; (**b**) roof angles between 7° and 27°.

The previous full-scale ASTM E1592 experimental study of an SSMR with a plastic saddle showed that three full-scale plastic saddle SSMRs failed at an average pressure of P=5.103 kPa, as shown in Table 5 [2]. To account for the failure wind speed of wind-clip-enhanced SSMRs, the average failure pressure of the strengthened SSMRs with wind clips was assumed to be 1.2077 times the average failure pressure of the full-scale plastic saddle SSMR test ($P_{WC}=P\times 1.2077$) on the basis of the lab-scale experimental results presented in Table 5. The failure wind speed vs. building height curves were obtained by considering the effects of three roof zones and roof angles on the basis of the ASCE 7-10 standards [34]. The wind velocity of a 3 s gust at eave height can be calculated from the uniform pressure measured from ASTM E1592 and the velocity pressure exposure coefficient, $K_{z}$, using the following equation:

Vh, 3sec=P0.613KzKztKdGCpIKz=2.01(zzg)2α for  15 ft≤z≤zgKz=2.01(15zg)2α for  z<15 ft

where *P* is the uniform pressure, $K_{zt}$ is the topographic factor, $K_{d}$ is the wind directionality factor, and $GC_{p}$ represents the pressure coefficient. The directional ($K_{d}$) and topographic ($K_{zt}$) factors were taken as 0.85 and 1, respectively, on the basis of a previous study [35]. Zone I represents the field area of the roof panel, and Zone II and Zone III are the edges and corners of the panel, respectively, as shown in Figure 11. On the basis of our assumptions of the averaged failure pressure for an SSMR reinforced with wind clips, the failure wind speed was computed for Zone II and III for strengthened SSMRs for the edges and corners of the roof when considering the roof height and angle in order to enhance the fragile sections of metal roofs that were observed in a previous study [35]. The computed failure wind speed at 10 m building height indicated that the performance of the SSMR was enhanced by about 6 m/s and 7 m/s in Zone II and Zone III, respectively, after reinforcement with wind clips.

## 5. Conclusions

Recently, renovated standing seam metal roofs (SSMRs) have been chosen for high-technology industrial buildings to increase the waterproofing without a self-drilling screw and to improve heat insulation with glass wool insulation material. On the basis of previous full-scale experiments involving wind-induced roof panel displacement and clip strain measurements that were conducted under cyclic wind loading and unloading methods based on the ASTM E1592 regulations, the major failure mechanism of renovated SSMRs was determined to be mid-clip rupture. By focusing on the mid-clip, plastic saddle, and roofing sheet components of the renovated SSMR system with respect to a concentrated load on the mid-clip part of metal roofing sheet, we conducted a lab-scale experimental and numerical investigations with the objective of evaluating and strengthening the structural performance of plastic saddle-type SSMRs under various loads and plastic saddle angles. The major observations and conclusions based on the lab-scale SSMR experimental and numerical tests can be summarized as follows:

⯀ A lab-scale renovated SSMR experimental study of fragile structural components was carried out to assess the effects of the governing factors and strengthening methods on mechanical performance. The mechanical responses of the SSMR were highly dependent on the saddle angle and loading speed. Gradual increases in the peak load and the displacement at peak load were observed as the loading speed increased. The overall mechanical performance of the tilted plastic saddle was degraded as the saddle angle increased.
⯀ Experimental results were utilized to calibrate the Johnson–Cook strength parameter for the SSMR finite element simulations. The calibrated JC model was applied to study the dynamic failure response of the lab-scale experiments under various loading speeds and saddle angles and was used to observe the dynamic response of large-scale SSMRs under dynamic wind loading conditions.
⯀ In the comparison between experimental tests with and without strengthened SSMRs, the strengthened SSMR exhibited a 20.7% improvement in mechanical performance. On the basis of the lab-scale strengthened SSMR test results, the failure wind speed of strengthened SSMR was computed on the basis of ASCE 7–10 standards by considering uplift zone effects and the height and angle of the roof by assuming that the corners and edges of the roof system were strengthened with wind clips. The computed failure wind speed was enhanced by about 6 m/s and 7 m/s at Zone II and Zone III, respectively, after reinforcement with wind clips.

The results of this study highlight the major factors influencing the structural performances of lab-scale renovated standing seam metal roofs by considering the loading speed, plastic saddle angle, and wind clip reinforcement. An improvement in the failure wind speed of SSMRs strengthened at the corners and edges of the roof was observed and computed to evaluate the overall structural capacity under various geometric profiles of the roof structure. Future studies are necessary to simulate the dynamic response of SSMRs with the Johnson–Cook strength model under dynamic loading conditions and various geometric profiles to extend the sustainability of their structures. A fragility analysis of SSMR should be carried out to estimate the wind speed–damage relationship for uplift wind loads. In addition to full-scale FE simulations, the necessity of geometry optimization of wind clamps should be investigated for the future study to maximize structural stability of SSMR.

## Figures and Tables

**Table 1:** Material components of the lab-scale SSMR test.

Roof System	Material	Geometry
Metal roofing sheet	Galvanized steel	W 325 × H 500 × T 0.7 mm
Saddle material	Plastic saddle	W 85 × H 235 × T 56 mm
Mid-clip	Mid-clip	L 96 × H 63 × W 24 × T 1.0 mm

**Table 2:** Test results of lab-scale SSMR specimens.

Test Specimen	Speed (mm/min)	Saddle Angle (°)	Failure Load (kN)	Average Failure Load (kN)	Test Specimen	Speed (mm/min)	Saddle Angle (°)	Failure Load (kN)	Average Failure Load (kN)
PS-v10-a0-1	10	0	5.440	5.251	PS-v500-a0-1	500	0	6.368	6.302
PS-v10-a0-2	10	0	5.271		PS-v500-a0-2	500	0	6.289	
PS-v10-a0-3	10	0	5.042		PS-v500-a0-3	500	0	6.250	
PS-v50-a0-1	50	0	5.460	5.432	PS-v10-a20-1	10	1.72	5.099	5.019
PS-v50-a0-2	50	0	5.394		PS-v10-a20-2	10	1.72	4.938	
PS-v50-a0-3	50	0	5.440		PS-v10-a20-3	10	1.72	5.019	
PS-v100-a0-1	100	0	5.360	5.462	PS-v10-a30-1	10	2.58	5.303	4.860
PS-v100-a0-2	100	0	5.453		PS-v10-a30-2	10	2.58	4.770	
PS-v100-a0-3	100	0	5.577		PS-v10-a30-3	10	2.58	4.508	
PS-v200-a0-1	200	0	5.646	5.666	PS-v10-a40-1	10	3.44	4.354	4.210
PS-v200-a0-2	200	0	5.708		PS-v10-a40-2	10	3.44	3.714	
PS-v200-a0-3	200	0	5.654		PS-v10-a40-3	10	3.44	4.562	
PS-v300-a0-1	300	0	5.929	6.024	PS-v10-a50-1	10	4.30	3.930	3.938
PS-v300-a0-2	300	0	6.001		PS-v10-a50-2	10	4.30	3.616	
PS-v300-a0-3	300	0	6.144		PS-v10-a50-3	10	4.30	4.268	

**Table 3:** Constitutive Johnson–Cook model parameters of the mid-clip [31].

	*E* (GPa)	ν	*A* (MPa)	*B* (MPa)	*n*	*C*	$\dot{\varepsilon_{ref}}$ s^−1^
Mid-clip	200	0.28	75	210	0.5176	0.0565	1

**Table 4:** Experimental results of lab-scale SSMR tests with wind clips.

Test Specimen	Speed (mm/min)	Failure Load (kN)	Average Failure Load (kN)
V10-1	10	5.440	5.251
V10-2	10	5.271	
V10-3	10	5.042	
Windclip-V10-1	10	6.433	6.342
Windclip-V10-2	10	6.250	

**Table 5:** Failure wind speed with and without wind clips at 10 m building height.

Test Specimen	Average Failure Pressure (kPa)	Failure Wind Speed θ°< 7° (m/s)	Failure Wind Speed 7° < θ< 27° (m/s)
Zone I	P=5.103	98.96	104.31
Zone II		73.76	75.90
Zone III		59.14	61.37
Zone II with wind clip	6.168 $(P_{WC}=P\times 1.2077$)	81.06	83.41
Zone III with wind clip		64.99	67.45

## Data Availability

Not applicable.
